# Single Neurons in the Insular Cortex of a Macaque Monkey Respond to Skin Brushing: Preliminary Data of the Possible Representation of Pleasant Touch

**DOI:** 10.3389/fnbeh.2016.00090

**Published:** 2016-05-24

**Authors:** Laura Clara Grandi, Marzio Gerbella

**Affiliations:** Department of Neuroscience, Physiology Unit, University of ParmaParma, Italy

**Keywords:** single neurons, perisylvian region, insular cortex, *Macaca mulatta*, pleasant touch, grooming

## Abstract

Pleasant touch may serve as a foundation for affiliative behavior, providing a mechanism for the formation and maintenance of social bonds among conspecifics. In humans, this touch is usually referred to as the caress. Dynamic caressing performed on the hairy skin with a velocity of 1–10 cm/s is perceived as being pleasant and determines positive cardio-physiological effects. Furthermore, imaging human studies show that affiliative touch activates the posterior insular cortex (pIC). Recently, it was demonstrated that pleasant touch in monkeys (i.e., sweeping in a grooming-like manner) is performed with velocities similar to those characteristics of human caress (9.31 cm/s), and causes similarly positive autonomic effects, if performed with velocity of 5 cm/s and 10 cm/s, but not lower or higher. Due to similarities between the human caress and non-human primate sweeping, we investigated for the first time whether single neurons of the perisylvian regions (secondary somatosensory cortex [SII] and pIC) of a rhesus monkey can process sweeping touch differently depending on the stimulus speed. We applied stimulation with two speeds: one that optimally induces positive cardio-physiological effects in the monkey who receives it, and includes the real speed of sweep (5–15 cm/s, sweep fast), and a non-optimal speed (1–5 cm/s, sweep slow). The results show that single neurons of insular cortex differently encode the stimulus speed. In particular, even the majority of recorded somatosensory neurons (82.96%) did not discriminate the two speeds, a small set of neurons (16.59%) were modulated just during the sweep fast. These findings represent the first evidence that single neurons of the non-human primates insular cortex can code affiliative touch, highlighting the similarity between human and non-human primates’ social touch systems. This study constitutes an important starting point to carry out deeper investigation on neuronal processing of pleasant sweeping in the central nervous system.

## Introduction

The sense of touch is a critical and necessary sense for our experience of the world in daily life, not only to haptically explore objects but also to communicate both negative and positive messages during social interactions (Hertenstein et al., 2006a,b). Human studies show that the affiliative touch involving the hairy side of the skin is a dynamic touch which activates specific unmyelinated fibers, the C-fibers, well-known as present not only in humans but also in various mammals, including non-human primates (Zotterman, 1939; Douglas and Ritchie, 1957; Bessou et al., 1971; Iggo and Kornhuber, 1977; Kumazawa and Perl, 1978; Johansson et al., 1988; Nordin, 1990; Vallbo et al., 1995; Morrison, 2012). These fibers are modulated by a dynamic touch performed on the hairy side of the skin with a specific speed of 1–10 cm/s, while they decrease their firing rate if the speed of stimulation is lower than 1 cm/s or higher than 10 cm/s (about 30 cm/s). Indeed, their curve of stimulation has the characteristic upside down *U-*firing shape with the maximal firing rate for the speed 1–10 cm/s (Vallbo et al., 1995; Löken et al., 2009; Morrison, 2012). This tactile stimulation perceived by means of C-fibers is pleasant for the subjects who receive it (Löken et al., 2009), and it was demonstrated to determine positive physiological effects toward the vagal modulation (Field et al., 1986; Tsao, 2007; Garnera et al., 2008; Russell et al., 2008; Billhult et al., 2009; Diego and Field, 2009; Lindgren et al., 2012; Field, 2014; Schroeder et al., 2014). Concerning the cortical processing, these fibers project to the superficial lamina I and inner lamina II of the spinal dorsal horn (Kumazawa and Perl, 1978; Light and Perl, 1979; Sugiura et al., 1986). Information conveyed by C-fibers are relayed, at higher levels, by the ventromedial posterior thalamic nucleus to the dorsal posterior insular cortex (pIC; Olausson et al., 2002, 2008a,b, 2010; Björnsdotter et al., 2009), to the orbitofrontal cortex (a key area for hedonic processing), to the postero-superior temporal sulcus (STS), the medial prefrontal cortex, the dorso-anterior cingulate cortex, and the pregenual anterior cingulate cortex (Kringelbach and Rolls, 2004; McGlone et al., 2007, 2012; Gordon et al., 2013; Ellingsen et al., 2014). These anatomical data are consistent with the idea that these fibers belong to the interoceptive system, and play a pivotal role in the encoding of the pleasantness and emotional component of interpersonal touch. Noteworthy that although to what extent these areas specifically process pleasant touch it remains largely unexplored, the insular cortex, primarily the posterior part, appears to have an especially important role (Olausson et al., 2002, 2010; Björnsdotter et al., 2009).

The affective touch has strong significance not only for humans but also for all social mammals, including non-human primates (Dunbar, 2010). Nevertheless, the knowledge about the mechanisms behind the codification of pleasant touch in monkeys is very poor. Indeed, there are no monkey studies exploring the neural correlate of the pleasantness of touch. Only recently, a role of C-fibers during pleasant sweeping movements associated with grooming, the most important social behavior among monkeys, was hypothesized (Dunbar, 2010). The sweeping can be described as the hand action that the agent monkey performs during grooming to move the hair of the recipient monkey in order to select an area of skin to be picked, for removing any ectoparasites and vegetation trapped in the fur. Thus, this action does not merely have a hygienic function, but also contains an affiliative meaning, as the caress in humans. Until now, only indirect evidence exists in support of this hypothesis. Brain lesion studies (Kling and Steklis, 1976) and single neurons recordings (Ishida et al., 2014) demonstrated that the inner perisylvian regions (secondary somatosensory cortex [SII] and pIC) seems to be involved in the encoding of the grooming behavior of monkeys. In terms of autonomic modulation, it is well known that grooming determines relaxation in monkeys. In particular, a decrement of the heart rate (HR) during received grooming (Boccia, 1989; Boccia et al., 1989; Aureli et al., 1999) and a reduction of the cortisol levels during both passive (Gust et al., 1993) and active grooming (Shutt et al., 2007) were demonstrated. Recently, it was also reported that both the single hand actions of grooming, picking (Grandi and Ishida, 2015) and sweeping (Grandi et al., 2015) determined similar autonomic effects in terms of the HR and heart rate variability (HRV). In particular, the sweeping that determined the decrement of HR and increment of HRV, and therefore of the vagal tone, was performed with speeds of 5 and 10 cm/s, which are similar velocities to those associated with human C-fibers’ activation. Furthermore, a pilot kinematic study showed that the velocity of real sweeping is 9.31 cm/s, and therefore it is in the range of the optimal velocity (1–10 cm/s) to activate the C-fibers in humans (Grandi et al., 2015). Finally, the positive autonomic effects were also reported in terms of body temperature changes. In fact, the sweep on the back of a male rhesus monkey occurring with a velocity in the range of 5–10 cm/s determined an increment of the nose skin temperature, parallel to the increment of the HRV and decrement of HR (Grandi and Heinzl, 2015). The increment of nose skin temperature could indicate the positive meaning of stimulation in non-human primates (Ioannou et al., 2015).

Because the pIC appears to have a key role for coding the affiliative interpersonal touch in humans (Olausson et al., 2002, 2008a,b, 2010), and for triggering social behavior in non-human primates (Kling and Steklis, 1976; Ishida et al., 2014; Jezzini et al., 2015), here we investigated the modulation of single neurons in the pIC and in the adjacent SII region of a macaque monkey during the above cited dynamic tactile stimulation considered pleasant for non-human primates: sweeping. We applied stimulation with two velocities: one that optimally induce positive cardio-physiological effects and includes the real speed of sweep (5–15 cm/s, sweep fast) and a non-optimal speed (1–5 cm/s, sweep slow). The present study represents the first evidence of the insular representation of such affiliative touch in non-human primates, and an indirect evidence of a modulatory role of C-fibers in the cortical coding of the pleasant touch.

## Materials and Methods

### Subject

**Figure 1:**
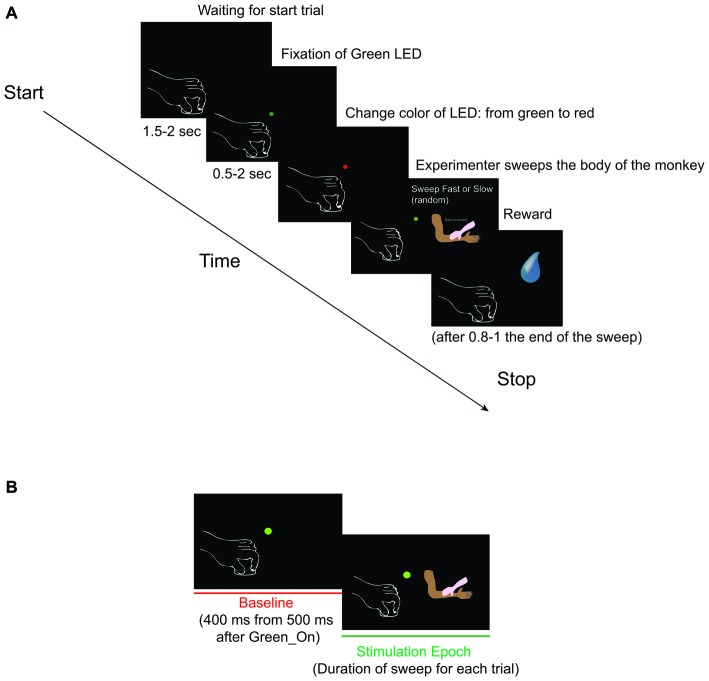
**(A)** Somatosensory task: Sweep Slow and Sweep Fast. The two sweeping were randomly applied, but for both the task was the following: (1) Green_On—Green_Off: the green light-emitting diode (LED) turned on for 0.5–2 s. We requested monkey to fixate the LED for all duration. (2) Red_On: the green LED switched to red and the experimenter moved the hand toward the monkey’s body part to be stimulated. We requested monkey to maintain the fixation. (3) Trg2_On—Trg2_Off: the experimenter touched the monkey (Trg2_On). The red LED switched off and the green LED was turned on until the sensory stimulation was finished (Trg2_Off). We requested monkey to maintain the fixation until the end of the sweeping. (4) Once the sweep had finished, the green LED switched off and after 0.8–1 s the monkey received drops of juice as a reward. After 1.5–2 s, the next trial began. From the end of stimulation to the start of the next trial we did not request the monkey to carry out any fixation task, but just to maintain the hands in their position. **(B)** The two epochs employed for statistical analyses. (1) Baseline: from 500 to 900 ms after Green_On (total of 400 ms); and (2) Stimulation: from Trg2_On to Trg2_Off. (For details see “Recording of Behavioral Events and Definition of Epochs of Interest” Section).

The experiment was performed on one 5-year-old male *Macaca mulatta* (5.5 kg). Since the monkey had been used as the subject of another previous electrophysiological experiment, he was already habituated to sitting on a primate chair, interacting with the experimenters and performing a fixation task, as described below (Figure 1A). Moreover, the head fixation system (Crist Instruments) and a circular (2 cm diameter) stainless steel chamber (Narishige, Japan) were implanted on the left hemisphere under general anesthesia (ketamine hydrochloride, 5 mg/kg i.m. and medetomidine hydrochloride, 0.1 mg/kg i.m.), followed by post-surgical pain medication. The study was performed by first recording on the left hemisphere, and then, once the recording sessions in that hemisphere were finished, the circular (2 cm diameter) stainless steel chamber (Narishige, Japan) was implanted on the right hemisphere. The correct position of both chambers to reach the interested deep brain area in order to record it was established based on the fMRI of that monkey’s brain. All surgical procedures were the same as described previously in Ishida et al. (2013). All experimental protocols were approved by the Veterinarian Animal Care and Use Committee of the University of Parma, and complied with European law on the humane care and use of laboratory animals. The monkey was kept in an individual primate cage consisting of a full metallic grid (Tecniplast S.p.A. Buguggiate, VA, Italy). The cage was 180 cm in height, 90 cm in width, and 120 cm in depth. In the middle of the vertical side of the cage, it was possible to insert one or two panels for the monkey to sit on. The litter was located immediately below the cage. In the bottom part of the cage two containers were located, one for water and the other for food, which were replenished by the experimenter. The monkey was provided with food and water once daily, typically in the morning. Inside the cage, the monkey had access to toys, mirrors, and swings. Furthermore, the monkey had visual, auditory, and olfactory contact with others and was able to touch and groom with the neighboring monkeys. The cage was in an air-conditioned room maintained at a consistent temperature of 25–26°C. The well-being and health condition of the monkey was constantly monitored by the institutional veterinary doctor of the University of Parma, Italy.

### Definition of Receptive Field

Each isolated neurons in SII and pIC was clinically tested in order to identify the property and the localization and the length of receptive field (RF). The clinical tests consisted of passive static and dynamic touch on all body parts, bilaterally. The touch was applied with the monkey’s eyes closed and open. Each neuron showing a clear passive RF was then tested with more structural task (see “Apparatus and Behavioral Paradigm” Section; Figure 1A).

### Apparatus and Behavioral Paradigm

During the recording sessions, the monkey had to perform a fixation task while receiving sensory stimulation from the experimenter, on the RF identified during clinical test (see “Definition of Receptive Field” Section). The task consisted of two passive dynamic somatosensory stimulations (sweeps), randomly applied to the RF of each neuron: (1) sweep with a speed of 1–5 cm/s (we defined this condition as sweep slow); and (2) sweep with a speed of 5–15 cm/s (we defined this condition as sweep fast). The stimulations were manually applied by the experimenter, who was trained to apply the stimulation through the two velocity ranges and with the same pressure irrespective of the speed. The exact applied velocity was calculated offline and confirmed.

The body part and the direction of stimulation selected to test each neuron were those that best elicited the neuron activity, as identified during clinical tests. The stimulation covered all lengths of the RF.

During the total length of the task, the monkey had to maintain the hand contralateral to the recording hemisphere on a metal cylinder (diameter 3 cm, height 2.5 cm) fixed to a plane close to the monkey’s body, and the ipsilateral one on the plane. A light-emitting diode (LED) was located in front of the monkey at a distance of 80 cm, which the monkey had to fixate for the duration of the task. The task (Figure 1A) consisted of:

1. Green_On—Green_Off: The green LED turned on for 0.5–2 s.
2. Red_On: The green LED switched to red and the experimenter moved the hand toward the monkey’s body part to be stimulated.
3. Trg2_On—Trg2_Off: The experimenter touched the monkey (Trg2_On). The red LED switched off and the green LED was turned on until the sensory stimulation was finished (Trg2_Off).
4. Once the sweep had finished, the green LED switched off and after 0.8–1 s the monkey received drops of juice as a reward. After 1.5–2 s, the next trial began. From the end of stimulation to the start of the next trial we did not request the monkey to carry out any fixation task, but just to maintain the hands in their position.

### Recording Techniques

We recorded from SII and from the pIC, in particular the disgranular insular cortex (dI) and the granular insular cortex (Ig), in both the left and right hemispheres. For each penetration, the correct depth was estimated on the basis of the properties in the different regions above, from the first neuronal activity detection in the convexity. The position of the chambers was decided based on the magnetic resonance of the monkey in order to reach more effectively the maximal possible length of the SII, dI and Ig. In the right hemisphere, the extracellular single unit recordings were carried out utilizing varnish-insulated tungsten microelectrodes (length 100 mm, impedance 1.5–3.0 MΩ at 1 kHz; FHC, USA), while we clinically tested the neurons in the recording site, each 300 μm.

In the left hemisphere, the neuronal recordings were performed by means of a 16 channel V-probe (@ Plexon). Both the V-probe and the tungsten microelectrodes were inserted vertically (90°) into the cortex through the intact dura matter via a hydraulic microdriver manipulator (Narishige, Japan). Each isolated single neuron in each of the 16 channels was first clinically tested. Then, following the first recording session, we moved down the V-probe in case the probe had not covered the entire area of interest, in order to conduct a second recording session. During the second recording session we considered solely the channels where we discriminated new single neurons and neurons not already recorded in the first session.

In both the left and right hemispheres, once we reached the white matter at the end of the recording sessions, we moved down the V-probe/tungsten electrode until we reached the next cortex, in order to physiologically confirm the recording site. The neuronal activity was recorded by a Plexon system. Then, both the analogic and digital signals acquired on-line were sent to a personal computer and stored for subsequent analysis, while the off-line analysis was conducted using a specific Offline Sorter Software (OmniPlex^TM^).

### Recording of Behavioral Events and Definition of Epochs of Interest

During the task, the starting and the switching off of each LED was monitored by LabView-based Software. Distinct contact-sensitive devices (Crist Instruments) were utilized to detect the maintaining of the hands’ position, and when the experimenter began and finished touching the monkey’s body. These devices provided a transistor-transistor logic (TTL) signal, which enabled the monitoring of the monkey’s performance. The eye position was monitored by an eye tracking camera (ISCAN Camera, ETL-200), with the eye position signals, together with the TTL events generated during task execution, being sent to the LabView-based Software so that the progress of the task could be monitored. Based on the TTL and eye position signals, the software enabled us to automatically interrupt the trial if the monkey broke fixation and/or the hands’ position. In such instances, no reward was delivered. The monkey always received the same amount of juice as a reward following the correct performance of each trial. Based on the digital signals related to the LED and the two events determined by the contact between the hand of the experimenter and the body of the monkey, we defined two different epochs of interest (Figure 1B) for statistical analysis of the neuronal responses: (1) Baseline: from 500 to 900 ms after Green_On (total of 400 ms); and (2) Stimulation: from Trg2_On to Trg2_Off. The time interval from 500 to 900 ms after the Green_On (total of 400 ms) was selected as the baseline, since this interval was situated after the trial had commenced and before the monkey had received the sensory stimulation. In this interval, the monkey did not see the hand of the experimenter since he had to fixate on the LED and did not know when and which kind of sweep he would receive. The time interval selected for the stimulation was the exact interval of the duration of sensory stimulation for each condition and each trial. Since the stimulation was manually performed by the experimenter, and thus without any strictly on-line control of the applied speed, prior to commencing any analysis we verified that the speed of each trial in each of the two conditions was performed with the velocity in the desired range; that is, 1–5 cm/s and 5–15 cm/s for the Sweep Slow and Sweep Fast, respectively. In order to calculate the speed, the cm range for each of the tested body parts was divided by the exact time of the respective stimulation. The time of stimulation for each trial was calculated by extracting the time of Trg2_Off from that of Trg2_On.

### Data Analyses and Classification of the Recorded Neurons

Single units were isolated on-line and off-line analysis was carried out utilizing a specific Offline Sorter Software (OmniPlex^TM^).

### Definition of Task-Related Neurons

Neurons were defined as being task-related if they significantly varied their discharge during a stimulation epoch in at least one of the two conditions in comparison to the baseline. The data’s normality and equality of variance (for each parameter in each condition) were verified with the Kolmogorov Smirnov and Levene tests, respectively. In order to identify the task-related neurons we performed repeated measures of 2 × 2 ANOVA (factors: Conditions and Epochs), followed by Bonferroni *post hoc* testing in the case of significant epochs and/or interaction condition × epochs’ effect (*p* < 0.05). The *post hoc* was executed to enable investigation of possible differences: (1) between the epochs in each condition; (2) between the conditions in each epoch; and (3) between the conditions independent of the epochs.

The neurons highlighting the epochs’ effect but not the statistical differences between the stimulation epochs of the conditions were classified as *speed unselective* (SU) neurons. The neurons showing the statistical difference between the stimulation epochs of the two conditions were instead defined as *speed selective* (SS) neurons. Among the second category, we identified them as *fast selective* if they showed the stimulation epoch of sweep fast that was statistically higher than the one of the sweep slow; and *vice versa* for the *slow selective* neurons.

The raster and histograms of the single unit data revealed the firing rate (spike/s) as normalized for the maximal value between the two conditions in each single unit. The alignment was the Trg2_On, and therefore when the experimenter began the stimulation of the monkey’s body.

### Correlation Analyses

In order to investigate if the neuronal discharge of SS neurons correlated with the velocity of the applied speed for each trial, or if they were selective for the range of velocity, we performed correlation analysis. As a control, we also conducted the same analysis for the SU neurons. Since both categories presented a linear correlation, even at a different percentage, we performed the Mann-Whitney U Test to compare the distribution of R-square.

### Population Analyses

Population analyses were performed on categories of neurons, classified on the basis of the results of the above-described analyses, and taking into consideration the single-neuron responses calculated as normalized activity (spike/s) in 20 ms bins, in the two tasks (sweep fast and sweep slow). The normalized activity for each neuron was calculated as follows: the mean activity was calculated every 20 ms bins in all the recorded trials of every experimental condition to be compared. Then, the absolute highest activity value was employed to divide the value of each single bin in all conditions (normalized mean activity, ranging from 0 to 1).

The histograms described the moving average of 60 ms of the normalized activity, for each of the two trials. The alignment was the moment when the experimenter touched the body of the monkey.

In order to investigate the bins where the two conditions significantly differed from one another in terms of population, we performed the 2-pairs *t*-test (*p* < 0.05) for each of the three populations. This took into account each 20 ms (one bin) from the Trg2_On, and therefore when the experimenter began the stimulation to the monkey’s body, until 2 s had elapsed. We selected 2 s as the maximal time to analyze the population data since the stimulation (both sweep fast and sweep slow) were completed within 2 s. A minimum of three bins were selected for significance. For the population analysis we did not consider the *slow selective* neuron in the population of SS neurons. All statistical analyses were carried out using Statistica Software (StatSoft).

### Anatomical Definition of the Recording Sites

In order to accurately identify the location of the recording sites, at the conclusion of the recording sessions and 10 days prior to sacrificing the monkey, electrolytic lesions (10 μA cathodic pulses, duration 10 s) were performed in both the hemispheres (Bonini et al., 2011). Specifically, for each hemisphere, known coordinates of the external borders of the recorded regions were selected and in each of them four lesions were made at different depths. Then, the monkey was deeply anesthetized with an overdose of sodium thiopental and perfused consecutively with saline, 3.5–4% paraformaldehyde, and 5% glycerol, prepared in a 0.1 M phosphate buffer and at pH 7.4, through the left cardiac ventricle. The brain was then blocked coronally on a stereotaxic apparatus, removed from the skull, photographed, and placed in 10% buffered glycerol for 3 days and 20% buffered glycerol for 4 days (Gerbella et al., 2014). Finally, it was cut frozen into coronal sections of 60 μm thickness, and the sections obtained will be processed with Nissl staining (0.1% thionin in 0.1 M acetate buffer, pH 3.7) in order to identify the electrolytic lesions.

Figures 6B and 6C present a section of the right hemisphere in which at least three penetrations located in the insular cortex and in the adjacent SII region were clearly visible. The section is taken at a mid-anterior level of the recorded region. The histological analysis confirmed that all the recording sites were located in the pIC or in the adjacent SII region.

## Results

We recorded a total of 223 task-related somatosensory neurons from both the right and the left SII, dI cortex and Ig cortex of one male rhesus monkey (*Macaca mulatta*). In particular, we recorded 107 neurons in the right hemisphere and 116 neurons in the left hemisphere. Since the recording sites were deep brain areas, the correct depth of each penetration was estimated on the basis of the physiological properties of the areas above the region of interest. Once the region of interest was reached, each isolated neuron was tested in order to identify the location and extension of its RF (see “Definition of Receptive Field” Section) and with the sweeping task (see “Apparatus and Behavioral Paradigm” Section; Figure 1A). Sensory neurons were considered as task-related only if they responded significantly during dynamic stimulation of their RFs during the task (see “Definition of Task-Related Neurons” Section). The location of neurons’ RFs where the sweeping was applied in the context of the task are presented in Table 1. All the isolated neurons had bilateral RFs, but the stimulation was always applied on the contralateral side.

**Table 1:** **Localization of receptive fields of task related neurons, SS and SU neurons**.

RF	Task related neurons	SS	SU
Mouth	71 (31,84%)	11 (28.95%)	60 (32.43%)
Hand	60 (26,91%)	13 (34.21%)	47 (25.41%)
Face	21 (9,42%)	4 (10.53%)	17 (9.19%)
Arm	48 (21,52%)	5 (13.16%)	43 (23.24%)
Upper body	3 (1,35%)	3 (7.89%)
Leg	15 (6,73%)		15 (8.11%)
Back	5 (2,24%)	2 (5.26%)	3 (1.62%)

Total	223	38	185

*The table shows the identified receptive field (RF) for the 223 task related neurons, 38 SS neurons and the 185 SU neurons. All RFs were located bilaterally but the stimulation was applied contralaterally*.

### Speed Selectivity of Task-Related Neurons

Task-related neurons were divided into two categories: speed selective (SS) and speed unselective (SU) neurons. The SS neurons showed a significant main effect of the factor Epoch and of the interaction between the factors Condition and Epoch. Therefore, they were modulated in one or both of the two sweeping stimulations, with the neuronal activity during one stimulation being significantly higher than the neuronal modulation during the other. The SU neurons only showed a significant main effect for the factor Epoch, thus being modulated during both the slow and fast sweeping, with no significant difference between the two.

**Figure 2:**
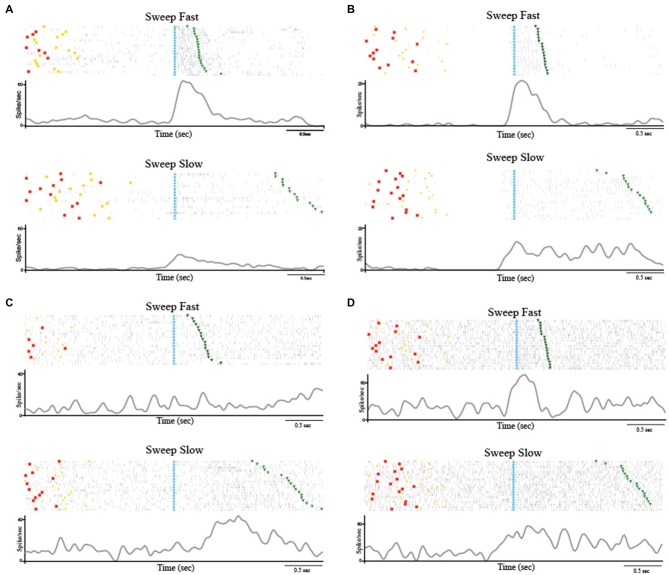
**(A)** Example of speed selective (SS) neuron (Fast Selective). The neuron was only modulated during the Sweep Fast. The neuron was recorded in the right hemisphere and the receptive field (RF) was located on the hairy side of the bilateral hand. **(B)** Example of SS neuron (Fast Selective). The neuron was modulated during both sweeping velocities, but the activity during the Sweep Fast was higher than that during the Sweep Slow. The neuron was recorded in the right hemisphere and the RF was located on the hairy side of the bilateral hand. **(C)** Slow Selective neuron, activated only during the Sweep Slow. The neuron was recorded in the left hemisphere and the RF was located on the bilateral hairy side of the mouth. **(D)** Example of speed unselective (SU) neuron. The neuron was modulated during both sweeping speeds, independently of the velocity. The neuron was recorded in the right hemisphere and the RF was located on the bilateral hairy side of the hand. The stimulation was performed on the contralateral side, from the distal (fingers) to the proximal part (near wrist). In **(A–D)** the histograms are aligned at the starting point of the stimulation, indicated by the light blue triangles. The end of the stimulation is indicated by the green triangles. The time between the red dots and yellow circles indicates the baseline, while the time between the light blue and green triangles represents the stimulation epoch. The histograms are normalized for the maximal spike/s value between the two conditions.

Among the 223 task-related neurons, 38 (17.04%) were SS and all of them were facilitated neurons. Table 1 shows the location of RFs of the SS neurons. Thirty-seven out of the thirty eight SS neurons were *fast selective*. Among the *fast selective* neurons, 14 were activated during both sweeping conditions, but the neuronal discharge during the stimulation epoch of the Sweep Fast was higher than the firing rate during the stimulation epoch of the Sweep Slow. Twenty-three out of the thirty seven *fast selective* neurons were only activated during the sweep fast condition, but not the sweep slow. Figure 2A shows an example of a SS neuron activated only during the Sweep Fast (*fast selective*), while Figure 2B shows an example of a SS neuron activated during both the fast and slow sweep, although the modulation during the sweep fast was higher than during the sweep slow (*fast selective*). Finally, just 1 out of the 38 was a *slow selective* neuron (Figure 2C), since it was only activated during the sweep slow and not during the sweep fast. One hundred and eighty-five of the two hundred and twenty three neurons (82.96%) were classified as SU neurons (Figure 2D) since they were modulated during both the conditions, but they did not discriminate between them. Table 1 shows the location of the RFs of SU neurons. Forty-eight out of one hundred and eighty five (25.94%) of the SU neurons were suppressed neurons, since their firing rate during the stimulation epoch decreased significantly relative to that during baseline, independently from the conditions.

These results highlight that a small set (*N* = 38) of task-related neurons discharge differently depending on the speed with which the passive touch stimulation was applied. Interestingly, just 1 out of 38 SS neurons showed higher modulation during slow stimulation (1–5 cm/s), while the remaining 37 neurons demonstrated selectivity for the fast stimulation (5–15 cm/s).

### Relationship Between Stimulation Speed and Firing Rate

The stimulus speed in each trial of the two tasks was not fixed as it was dependent on the experimenter’s movement, ranging from 1 to 5 cm/s during the sweep slow and from 5 to 15 cm/s during the sweep fast. In order to investigate more precisely the relation between the firing rate of each trial and the real applied velocity, we performed a trial-based correlation analysis (see “Correlation Analyses” Section) that considered the firing rate (spike/s) during the stimulation epoch and the speed (from 1 to 15 cm/s), independently from the condition (fast or slow) the trial was associated with.

The analysis evidenced that all the 38 SS neurons display statistically significant linear correlation between the speed of stimulation and the firing rate (spike/s). Among the SU neurons, only a minor part showed a significant correlation (21 out of 185, 11.35%).

The results indicate that SS neurons show a stronger relationship between firing rate and stimulus speed than SU neurons (*χ*^2^ = 17.26; *df = *1; *p* < 0.001).

**Figure 3:**
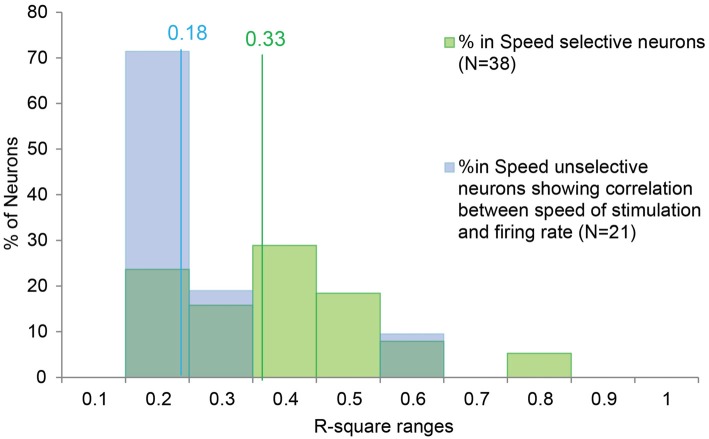
**Distribution of the R-square values of the SS and SU neurons showing significant correlation between the speed of the stimulus and the firing rate.** The lines indicate the median value for the SS (in green) and SU neurons (in blue). In order to investigate the potential similarity between the speed/firing rate correlation of SS neurons and the correlation showed by the SU neurons, we compared the distribution of R-square obtained in the two groups. The Mann-Whitney U Test indicates that the R-square of the SS neurons (median = 0.33) is higher (*p* < 0.001) than the R-square of the SU neurons (median = 0.18), thus speed variation account for a greater part of the variability of SS than SU neurons discharge.

Moreover, in order to better investigate the potential similarity between the speed/firing rate relationship among SS and SU neurons, we compared the distribution of R-square obtained in the two categories (Figure 3). The Mann-Whitney U Test indicated that the R-square of the SS neurons (median = 0.33) is higher (*p* < 0.001) than the R-square of the SU neurons (median = 0.18), thus speed variation account for a greater part of the variability of SS than SU neurons discharge.

### Population Analyses

**Figure 4:**
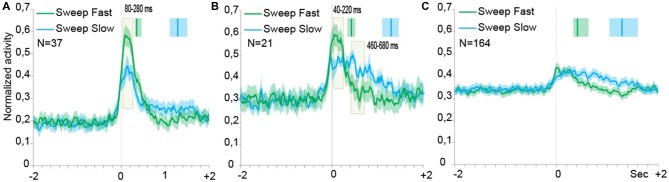
**Time course of the activity of neuronal populations aligned (gray line) on the tactile stimulation onset in the both conditions, Sweep Fast and Sweep Slow.** Green lines indicate the time course of activity during Sweep Fast, while blue lines represent the time course of activity during Sweep Slow. On the *Y* axis is represented the normalized activity of the neuronal populations, while on the *X* axis is represented the time (sec). **(A)** The population of the SS neurons is activated stronger during the Sweep Fast compared with the Sweep Slow, in particular from 80 ms after alignment to 280 ms. **(B)** The population of the SU neurons showing correlation between the speed of stimulation and firing rate. From 40 ms after the alignment to 220 ms, the population is activated stronger during the Sweep Fast than Sweep Slow, while from 460 to 680 ms the activity is higher during the Sweep Slow than during the Sweep Fast. **(C)** The population of the SU neurons that did not show correlation between the speed of sweeping and the firing rate. The population does not discriminate between the speed of stimulation and the two tasks do not differ from each other. In **(A–C)** the activity is in alignment when the experimenter touched the monkey’s body. The median times of the end of sweep fast and sweep slow are indicated with the green and blue markers, respectively, above each population plot. Shaded areas around each marker represent the 25th and 75th percentile times. In **(A,B)** the light green shaded regions indicate the period in which the paired samples *t*-tests evidence a significant separation of the two curves (*p* < 0.05).

Population analyses were performed in order to explore the time course and intensity of the population activity by taking into account three different groups of neurons, that is: (1) SS neurons, and SU neurons; (2) with; and (3) without speed/firing rate significant correlation. Figures 4A–C describe the results of the population analyses (see “Population Analyses” Section). The activation profile of *SS* neuronal population (Figure 4A) showed that these cells were activated during both types of sweeping but the activation was higher during the sweep fast than the sweep slow, particularly from 80 to 280 ms after the alignment point (stimulus onset).

Figure 4B shows the activation profile of SU neurons endowed with significant speed/firing rate correlation at the single neuron level. Similarly to the neuronal population of Figure 4A, these neurons discharged stronger during sweep fast relative to sweep slow from 40 to 220 ms after the alignment point, but the Sweep Slow induced a sustained discharge stronger than that during sweep fast later on from 460 to 680 ms after the alignment.

Moreover, from the profile of the modulation it is possible to notice that the modulation during the Sweep Fast and Sweep Slow began from the alignment, but unlike the SS population the activity did not drop at a similar timing. Instead, during the Sweep Fast the activity decreased approximately in a similar manner as the activity during the Sweep Fast of the SS population. During the Sweep Slow the activity remained stable for the entire length of the condition. Therefore, the population was modulated during both conditions for the full duration of the stimulation, from the alignment until after 2 s.

Figure 4C shows the activation profile of SU neurons with no significant speed/firing rate correlation at the single neuron level. The results evidenced the lack of any statistically significant difference between the response profile associated to the two sweeping conditions.

### Histological Analyses Confirmed the Recording Sites

**Figure 5:**
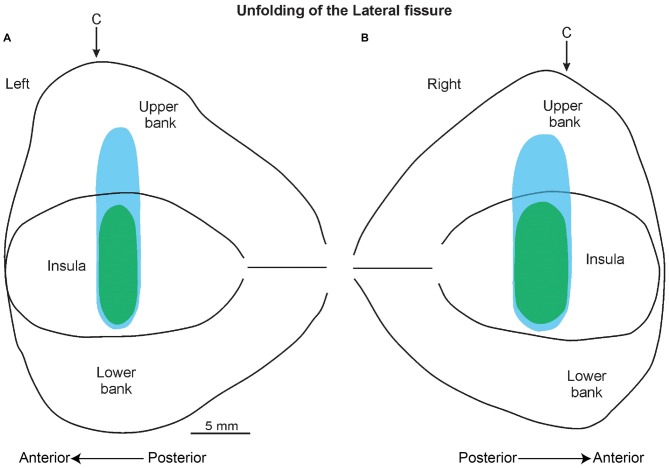
**A 2D reconstruction of the lateral fissure of the left (A) and right (B) hemisphere, aligned to the middle of the insula; the continuous lines mark the lips of the sulcus, the border of the insula with the upper and lower bank of the sulcus, and the fundus.** The blue area indicates the recorded region related to the SU neurons, while the green area indicates the recorded area of the SS neurons. The arrow marks the rostral-most level of the central sulcus (C).

**Figure 6:**
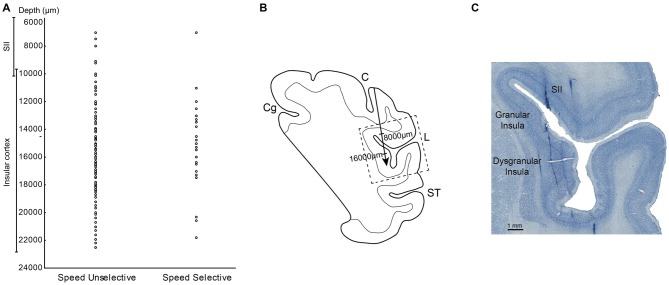
**(A)** Distribution of the SS and SU neurons in relation of the depths. **(B)** Drawing of the section in which the dashed box indicates the location of the photomicrograph in **(C)**. The arrow indicates the depth of track showed in **(C). (C)** High power photomicrograph of Nissl section from the right hemisphere in which it is possible to identify tracks of the recording electrode through the insular cortex and SII region. Abbreviations: L, lateral fissure; SII, secondary somatosensory cortex; ST, temporal sulcus; C, central sulcus.

The histological analyses based on the location of the electrolytic lesions, confirmed that the recorded sites, as expected from magnetic resonance data, are virtually all located in the pIC and in the adjacent SII region (Figures 6B,C). Figure 5 shows an unfolded map of the lateral fissure of both the left and the right hemisphere in which the blue and green areas represent the whole recorded region. In antero-posterior terms, this region starts from about the rostral-most part of the central sulcus and extends posteriorly from this point for 5 mm.

The measurements of the depth of each penetration revealed that the SS task related neurons appear virtually all located in the insular region. In fact, almost all these neurons were recorded at a depth compatible with that of the insula, that is from 11 to 21.8 mm below to the convexity (Figure 6A). In contrast, the SU neurons, since involving a larger sector, extending from 7 mm to 22.5 mm of depth appear located not only in pIC but also in SII (Figure 6A).

**Figure 7:**
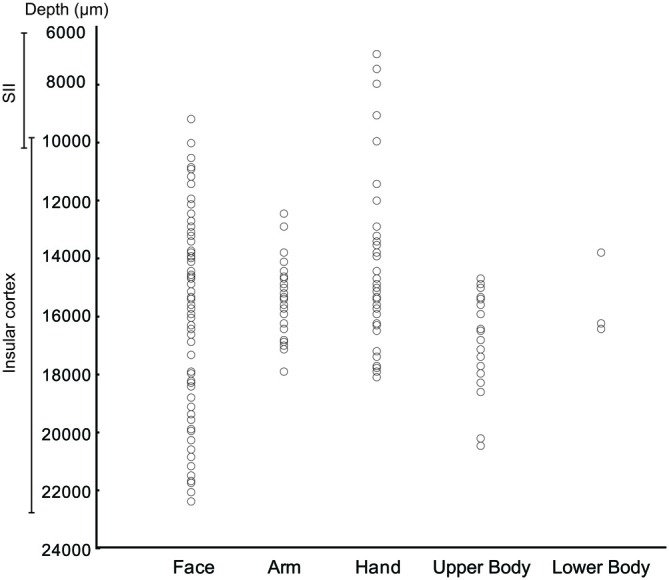
**Distribution of the task related neurons in relation to the localization of RFs**.

The analysis of the representation of the different RFs of both SS and SU neurons showed that the face representation tended to extend, in most of the recorded sites, more ventrally respect to the other RFs (Figure 7). In this analysis the RFs of mouth, cheek, and face were merged in a unique RF, that is the face, whereas the RFs of back and upper body were merged in the upper body RF, since back RF involved especially the dorsal part of the posterior body.

## Conclusion and Discussion

Due to the key role of the pIC in the coding of affiliative interpersonal touch in humans (Olausson et al., 2002, 2008a,b, 2010), and its hypothetical involvement in social behavior in non-human primates (Kling and Steklis, 1976; Ishida et al., 2014; Jezzini et al., 2015), here we investigated for the first time the activity of single neurons of the dysgranular and granular parts of this region (dI and Ig, respectively) of the macaque monkey during a dynamic tactile stimulation, considered pleasant for non-human primates, that is, sweeping. Sweeping is a common dynamic hand action that monkeys perform during grooming to move the hair and identify the skin sites to be picked in order to remove ectoparasites or vegetation trapped within. Even though grooming has primarily a hygienic function, it has been hypothesized that this behavior has also a social role (Maestripieri, 1993; Dunbar, 2010; McFarland and Majolo, 2011). The social function hypothesis is supported in part by studies demonstrating the positive autonomic effects of grooming (Boccia, 1989; Boccia et al., 1989; Aureli et al., 1999; Dunbar, 2010). In fact, the induced relaxation could be considered an indirect evidence that grooming creates social bonds among conspecific belonging to the same social group. Even though numerous behavioral studies supporting the social function of the grooming, there are no evidence related the real mechanisms at the basis of the affiliative role of this behavior. However recently, Dunbar (2010) supposed that the positive autonomic effects of grooming could be not due from this behavior *per se* but referable to the sweeping. In this respect it is noteworthy that sweeping activates specific unmyelinated fibers, the C-fibers (Dunbar, 2010) for which a specific role in encoding pleasant touch was supposed (Vallbo et al., 1995; Löken et al., 2009; Morrison, 2012).

Although, as of today, it remains largely unexplored to what extent specific cortical areas receive the somatosensory information conveyed by these fibers and thus how the cortex process the pleasant touch, human studies suggest that the insular cortex, primarily the posterior part, appears to constitute a crucial node for encoding this sensation (Olausson et al., 2002, 2010; Björnsdotter et al., 2009). An intriguing hypothesis came taking together these observations: as well the purely sensory cortices (SI and SII) are involved in the encoding the discriminative aspect of the touch, pIC could have a crucial role in the processing the emotional counterpart of this sensation. To test this hypothesis, in the present study we investigated the role of single neurons of SII and of the pIC during sweeping. In detail, we applied the sweeping with two speeds: one that optimally (5–15 cm/s, sweep fast) induce positive cardio physiological effects on the monkey that receive it (Grandi and Heinzl, 2015), and includes the real speed of sweep (Grandi et al., 2015) and a non-optimal speed (1–5 cm/s, sweep slow; Grandi et al., 2015).

We recorded a total of 223 task-related neurons, 38 of which were categorized as SS (SS; 17.04%), and 185 as SU (SU; 82.96%). The majority of the task-related neurons were SU neurons since they were modulated during the tactile stimulation, independently of the applied speed. Thirty seven out of thirty eight SS neurons coded for the fast speed (5–15 cm/s) since they were preferentially activated when sweeping was performed in the range of 5–15 cm/s. Just 1 of the 38 SS neurons discharged specifically when the speed was lower than 5 cm/s. The firing rate of all the SS neurons was correlated with the applied speed. Although the SU neurons did not discriminate the speed, a small percentage of them showed a positive correlation between their firing rate and the applied speed, even though this correlation appears to be significantly lower than that of the SS neurons. Moreover, even at the single neuron level this set of SU neurons endowed with a significant correlation did not discriminate between the two ranges of velocities, they did so at the population level. Finally, the SU neurons showing differential activity between the two speeds at the population level, but not at the single neurons’ level, and the correlation between their firing rate during the sweeping and the applied speed, could represent an intermediate population of neurons. The analyses of the locations of the recorded sites showed that, nevertheless the SU neurons are located in both the pIC and the adjacent SII region, the SS neurons appear almost at all locations in the posterior insula. Furthermore, the analysis of the depth in which both the task related neurons are recorded in relation to their RFs, showed that the representation of the face tended to extend ventrally to the representation of the hand/arm, in complete agreement with the somatotopy observed in previous functional studies and connectional investigations (Schneider et al., 1993; Caruana et al., 2011; Jezzini et al., 2012, 2015; Gerbella et al., 2014). Considered as a whole the insular region here investigated is a node of a network of frontal, cingulate, temporal, and parietal areas and limbic subcortical structures, and thus is a site of integration of sensory, emotional, motor, and social information (Mesulam and Mufson, 1982a,b; Chikama et al., 1997; Ongür et al., 1998; Stefanacci and Amaral, 2002; Barbas, 2007; Gothard et al., 2007; Grabenhorst and Rolls, 2011; Padoa-Schioppa and Cai, 2011; Jezzini et al., 2015). In particular, the connections with the prefrontal and the orbitofrontal cortex, the amygdala, the ventral striatum, the mediodorsal nucleus of the thalamus, the lateral hypothalamus, and the ventral tegmental area could represent the neural substrate for, on one side encoding the emotional aspects of sensory stimuli and integrating them with reward and memory information and on the other side for triggering the vegetative responses (as the decrease in the heart rhythm; see also below) accompanying the skin brushing/pleasant touch. Summarizing, the aforementioned anatomical data supporting the idea that our insular sector has, besides a direct involvement in the perception of sensory emotional and social stimuli, also a direct role to elicit the appropriate behavioral and visceromotor responses in line with the notion that the insula is endowed with an enactive component intrinsic to each social and emotional behavior (Di Cesare et al., 2015; Jezzini et al., 2015).

Since the majority of SS neurons encode for the optimal speed (5–15 cm/s), the present study represents the first single neurons’ evidence of the role of insular cortex in the encoding of pleasant touch in non-human primates. In a recent review, Dunbar (2010) proposed that the soft touch arising from sweeping might activate the C-fibers. Human studies demonstrated that C-fibers are activated by the interpersonal pleasant touch performed on the hairy skin with the speed of 1–10 cm/s. This stimulus is perceived as pleasant by the subject who receives it and as a consequence, it can determine a positive physiological effect (e.g., the decrement of the HR) and modulates specific brain areas (such as the insular cortex) involved in the emotional processing of the peripheral somatosensory stimulation (Björnsdotter et al., 2009; Löken et al., 2009; Morrison et al., 2011).

Our results are in line with existing mentioned human imaging studies (McGlone et al., 2007; Olausson et al., 2010; Gordon et al., 2013). Nevertheless, our preliminary data showed that single neurons of the insular cortex are modulated differently in relation to touch performed at speeds of 1–5 cm/s (slow) or 5–15 cm/s (fast), even if both velocities are within the 1–10 cm/s range. The discrepancy between the human results that highlighted that stimulation at 1–10 cm/s activated the insular cortex, and our results could be explained by the different hairy skin properties of the monkey, which presents as thick fur in comparison to humans, and therefore suggests a possible difference in the density of the C-fibers. Further studies will be necessary to investigate in greater depth the single neurons’ activity in the insular cortex and SII during sweeping, and consequently to the direct activation of C-fibers. Studies in that direction would investigate more precise the relationship between the modulation of single neurons of insular cortex and the speed of stimulation. We hypothesize that the action of sweeping with a robotic device perfectly maintains the same speed, trial after trials, providing a precise understanding of how the single neuronal activity is modulated by different speed and a comprehension of how real biological sweeping differs from the artificial one made by a robotic device (Shokur et al., 2013). Another interesting issue could be to investigate the single neuron activity during sweeping observation in order to address the possibility of presence of mirror-like activity related to touch (Blakemore et al., 2005; Schaefer et al., 2013).

The tactile stimulation modulating the C-fibers, and therefore the vagal tone and the insular cortex, is employed as a therapy in patients suffering from depression and chronic pain, in cancer patients undertaking invasive therapy, and also to reduce the stress and increase the wellbeing of healthy people (Field et al., 1986; Tsao, 2007; Garnera et al., 2008; Russell et al., 2008; Billhult et al., 2009; Diego and Field, 2009; Lindgren et al., 2012; Field, 2014; Schroeder et al., 2014). In the same manner, here we demonstrated that human sweeping modulated the monkey insular cortex. Moreover, the positive physiological effects of sweeping were shown (Grandi and Ishida, 2015; Grandi et al., 2015). Due to these neuronal modulations, autonomic responses and the affiliative value of sweeping and grooming for monkeys, human sweeping could have the potential to reduce the stress of single-housed caged experimental non-human primates, in addition to human grooming (Grandi and Ishida, 2015), with this being recently proposed as a method to improve the welfare of experimental animals (Reinhardt and Reinhardt, 2008).

Moreover, recently (Shokur et al., 2013), it was demonstrated that, in monkey, primary somatosensory and primary motor neurons respond to the observation of an avatar arm being brushing when the stimulation was preceded by the physical touch. Therefore, the physical touch determines the illusion that the avatar arm being integral part of own body, as the rubber hand illusion in humans. This study, since investigating the effect of brushing in terms of expanding of body schema, appears especially important for the development of neuroprostheses and artificial limbs. In the respect of the present results further investigations using virtual sweeping, could be useful to understand if and how the body schema change in relation to affiliative sensory stimulation, in order to test, in last resort, the possibility to implement pleasant touch as therapy for patients with prosthesis.

Taken together, the data available up to now and the results presented here support the hypothesis that the sweeping motion: (1) is performed at a speed similar to that which activates human C-fibers; (2) determines similar positive autonomic effects evoked by the C-fibers’ activation in humans; and (3) is coded at single neuron level in the same brain regions involved in the processing of the gentle caress in humans. Finally, notwithstanding that the present results were obtained from only one monkey, and so caution is required in the interpretation, our findings are the first evidence highlighting the role of insular cortex in the encoding of pleasant touch in non-human primates. The study highlights the similarity between human and non-human primates’ social touch systems, and can be considered as an important starting point in order to investigate in greater depth pleasant sweeping at the central nervous system level.

## Author Contributions

LCG: performed the experiment, designed the experimetal tasks, analyzed neuronal data, analyzed histological data, wrote article. MG: analyzed histological data, wrote article.

## Conflict of Interest Statement

The authors declare that the research was conducted in the absence of any commercial or financial relationships that could be construed as a potential conflict of interest.
